# Erythrodiol, an Olive Oil Constituent, Increases the Half-Life of ABCA1 and Enhances Cholesterol Efflux from THP-1-Derived Macrophages

**DOI:** 10.3389/fphar.2017.00375

**Published:** 2017-06-13

**Authors:** Limei Wang, Sarah Wesemann, Liselotte Krenn, Angela Ladurner, Elke H. Heiss, Verena M. Dirsch, Atanas G. Atanasov

**Affiliations:** ^1^Department of Pharmacognosy, University of ViennaVienna, Austria; ^2^Department of Pharmacology, Qingdao University School of PharmacyQingdao, China; ^3^Department of Molecular Biology, Institute of Genetics and Animal Breeding of the Polish Academy of SciencesJastrzebiec, Poland

**Keywords:** olive oil, erythrodiol, ABCA1, cholesterol efflux, protein degradation

## Abstract

Cholesterol efflux (ChE) from macrophages is an initial step of reverse cholesterol transport (RCT). The ATP-binding cassette transporter A1 (ABCA1) is a key transporter for ChE and its increased expression is regarded to attenuate atherosclerosis. Thus, the identification and characterization of molecules raising ABCA1 and thereby stimulating ChE is of pharmacological relevance. In this study, we tested dietary compounds from olive oil for their capacity of enhancing cellular ABCA1 protein level. We identified erythrodiol (Olean-12-ene-3β,28-diol) as an ABCA1 stabilizer and revealed its positive influence on ChE in THP-1-derived human macrophages. Among the nine tested compounds from olive oil, erythrodiol was the sole compound raising ABCA1 protein level (at 10 μM). None of the tested compounds impaired viability of THP-1 macrophages from 5 to 20 μM as determined by resazurin conversion. Western blot analyses of key membrane transporters contributing to ChE showed that the protein level of ABCG1 and scavenger receptor class B member 1 (SR-B1) remain unaffected by erythrodiol. Besides, erythrodiol (10 μM) did not influence the mRNA level of ABCA1, ABCG1, and SR-B1, as determined by quantitative reverse transcription PCR, but significantly inhibited the degradation of ABCA1 as evident by an increased half-life of the protein in the presence of cycloheximide, an inhibitor of *de novo* protein synthesis. Therefore, erythrodiol promotes ChE from THP-1-derived human macrophages by stabilizing the ABCA1 protein. This bioactivity makes erythrodiol a good candidate to be further explored for therapeutic or preventive application in the context of atherosclerosis.

## Introduction

The transport of accumulated intracellular cholesterol from peripheral cells (such as macrophages) to extracellular lipid-poor apolipoprotein (apo) A1 or high-density lipoprotein (HDL) is called cholesterol efflux (ChE), which represents the initial step of reverse cholesterol transport (RCT). The process of RCT counteracts the development of atherosclerosis by transporting peripheral cholesterol to the liver for excretion into the bile and ultimately the feces (Tall et al., 2001; Ohashi et al., 2005; Cuchel and Rader, 2006; Duffy and Rader, 2006). Growing evidence indicates that the trans-membrane transporter ABCA1 is a key molecular player implied in cardiovascular disease (CVD), the leading cause of deaths worldwide. In the context of atherosclerotic plaque formation, ABCA1 combats CVD by transporting intracellular cholesterol out of peripheral macrophages, thereby inhibiting the transformation of macrophages into foam cells (Oram, 2003; Madamanchi et al., 2005). Besides the predominant ABCA1-mediated ChE, ABCG1- and SR-B1-mediated ChE are also important pathways for ChE and they jointly contribute to most of the ChE from macrophages (Phillips, 2014). It is widely believed that intracellular free cholesterol transported via ABCA1 predominantly binds to apoA1, while free cholesterol effluxed through ABCG1 will bind to HDL to enter subsequent steps of RCT for ultimate excretion (Duffy and Rader, 2006; Phillips, 2014; Westerterp et al., 2014).

Olive (*Olea europaea* L.) oil is a major source of fat in the Mediterranean diet. The most abundant component of virgin olive oil is triacylglycerol (~99%). Further, components include i.e., free fatty acids, mono-, and diacyl-glycerols, and an array of lipids such as hydrocarbons, sterols, aliphatic alcohols, tocopherols, and pigments (Boskou and American Oil Chemists' Society, 2006). Uvaol, erythrodiol, maslinic acid, oleanolic acid, and betulinic acid are the main triterpenic compounds found in olive fruits, leaves, and oil (Covas et al., 2006b; Guinda et al., 2010). Among them, erythrodiol and uvaol could reach over 600 mg/kg oil in pomace olive oil (Canabate-Diaz et al., 2007). Olive oil phenolic components were mainly investigated for potential effects on inflammatory processes related to atherosclerosis since they are powerful antioxidants (Covas et al., 2006b; Covas, 2008). A randomized trial involving 200 healthy young men consuming daily 25 mL olive oil with different phenolic content for 3 weeks showed that the polyphenol content of olive oils was positively associated with increased high-density lipoprotein (HDL) cholesterol level and decreased serum markers of oxidation (Covas et al., 2006a; Covas, 2008). Besides, virgin olive oil is proven to be more effective than refined olive oil in terms of reducing the inflammatory status in a subsample of 28 volunteers with coronary heart disease (CHD; Fito et al., 2008), probably because refined oils do not have a significant content of polyphenols (Tripoli et al., 2005).

**Figure 1:**
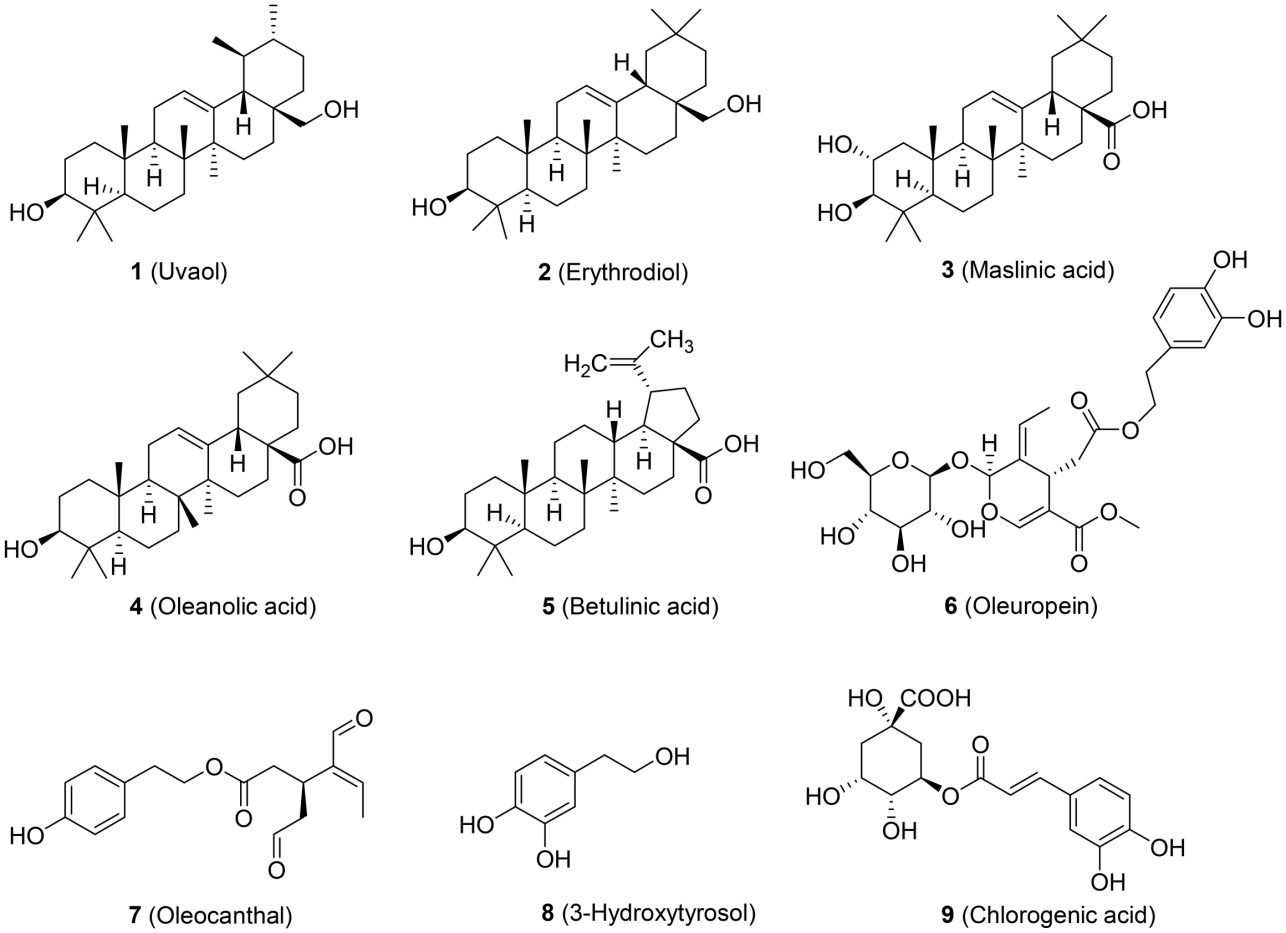
Chemical structure of the tested olive oil components.

Previous epidemiological studies pointed to a direct correlation between the Mediterranean diet and a lower incidence of CVD (Hertog et al., 1993). For example, ChE from human monocyte-derived macrophages (HMDM) isolated from healthy volunteers who consumed extra-virgin olive oil for 12 weeks was significantly increased by 44% compared to control. ABCA1 and ABCG1 mRNA transcription was significantly increased by 16.08 and 35.79%, respectively (Helal et al., 2013). Further, studies using polyphenol-rich olive oil revealed an increased gene expression of ABCA1, SR-B1, and PPAR receptors in white blood cells from 13 pre-hypertensive patients who consumed 30 ml of olive oils with 961 mg/kg polyphenol content in a randomized, controlled, cross-over trial (Farras et al., 2013). However, the specific compounds of olive oil responsible for its potential to combat CVD remain elusive. In our study, we have chosen the most abundant triterpenic compounds (uvaol, erythrodiol, maslinic acid, and betulinic acid) of olive oil and representative polyphenols (oleupropein, oleocanthal, 3-hydroxytyrosol, and chlorogenic acid; Figure 1) to investigate their potential to enhance ABCA1 protein level. Our results revealed that erythrodiol (Olean-12-ene-3β,28-diol), representing a reference compound for the analytical determination of olive oil, enhances ChE and concentration-dependently increases the levels of ABCA1 protein by inhibiting its degradation.

## Materials and methods

### Chemical reagents

Phorbol 12-myristate 13-acetate (PMA), water soluble unesterified cholesterol, apolipoprotein (apo) A1, resazurin sodium salt, cycloheximide, and digitonin were purchased from Sigma-Aldrich (Vienna, Austria). Pioglitazone was obtained from Molekula (Munich, Germany). [^3^H]-cholesterol (1 mCi, 37 MBq) was provided by Perkin Elmer Life Sciences (Vienna, Austria). The olive oil-derived compounds (uvaol, erythrodiol, maslinic acid, oleanolic acid, betulinic acid, oleuropein, 3-hydroxytyrosol, and chlorogenic acid) were supplied by Sigma-Aldrich (Vienna, Austria). Oleocanthal (>80% purity) was isolated from olive oil as recently described (Adhami et al., 2015) and was and kindly provided by Prof. Liselotte Krenn (University of Vienna). Bovine serum albumin (BSA-fatty acid free) was from Roth (Karlsruhe, Germany). All tested compounds were dissolved in DMSO, aliquoted and stored at −20°C until use. An equal amount of DMSO was always tested in each condition in all experiments to assure that the solvent vehicle does not influence the results.

### Cell culture and differentiation

Human THP-1 monocytic cells were obtained from ATCC® and cultured in T175 flasks with phenol red Roswell Park Memorial Institute (RPMI) 1640 medium (Lonza, Switzerland) supplemented with 2 mM glutamine, 100 U/mL benzylpenicillin, 100 μg/mL streptomycin, and 10% fetal bovine serum (FBS, Lofer, Austria). Cells were maintained at 37°C with 5% CO_2_ in a humidified atmosphere. The cultured THP-1 monocytes did not exceed 0.8 × 10^6^ per mL to assure viability higher than 95%. When needed, THP-1 cells were seeded at a density of 0.2 × 10^6^ per mL in 6-, 96-, or 24-well-plates for different experiments with 200 nM PMA for 72 h to allow differentiation into macrophages.

### Resazurin conversion assay

Resazurin conversion assay was performed as previously described (Wang et al., 2016). THP-1 macrophages were treated with the respective compounds (uvaol, erythrodiol, maslinic acid, oleanolic acid, betulinic acid, oleuropein, oleocanthal, 3-hydroxytyrosol, chlorogenic acid) at concentrations of 5, 10, and 20 μM for 24 h. After the incubation period, macrophages were washed with PBS and incubated with resazurin for another 4 h. Fluorescence (Ex/Em = 535/580 nm) of the generated resorufin was quantified as a measure of cell viability.

### Western blot analysis

THP-1 cells were seeded at a density of 0.2 × 10^6^ per mL in 6 well-plates with 200 nM PMA for 72 h to allow differentiation into macrophages. For screening of nine olive oil components for putative effects on ABCA1 protein expression, differentiated THP-1 macrophages were treated with the respective compounds (Figure 1) at 10 μM for 24 h. A PPARγ agonist, pioglitazone (10 μM), was used as positive control to augment ABCA1 expression. For testing, if erythrodiol is increasing ABCA1 protein level in a concentration-dependent manner, THP-1 macrophages were incubated with erythrodiol at different concentrations (1, 2.5, 5, 10, and 15 μM) for 24 h. For testing the effect of erythrodiol on ABCG1 and SR-B1 protein level, THP-1 macrophages were incubated with solvent vehicle control (DMSO), erythrodiol (10 μM), and pioglitazone (10 μM) for 24 h. To test the effect of erythrodiol on the ABCA1 protein degradation rate, differentiated THP-1 macrophages were treated with 10 μM erythrodiol or solvent vehicle control (DMSO). After 24 h incubation, cells were treated with 140 μM cycloheximide and lysed at the indicated time points (0, 20, 40, and 60 min). In general, cells were lysed using NP40 lysis buffer containing 150 mM NaCl, 50 mM HEPES (pH 7.4), 1% NP40, 1% protease inhibitor Complete™ (Roche), 1% phenylmethylsulfonyl fluoride (PMSF), 0.5% Na_3_VO_4_, and 0.5% NaF for 30 min at 4°C on ice. Macrophages were scraped from the plates and centrifuged at 16,060 × g for 30 min at 4°C. Protein concentration in cell lysates was determined using the Bradford assay. Western blot analysis was performed as previously described (Wang et al., 2015) using primary antibodies against ABCA1, ABCG1, and SR-B1 (all obtained from Novus Biologicals; Vienna, Austria). The anti-actin antibody as acquired from MP biologicals (Illkirch, France). HRP-linked anti-rabbit IgG secondary antibody was purchased from New England Biolabs (UK), and horseradish peroxidase conjugated goat anti-mouse secondary antibody from Upstate (Millipore, Vienna, Austria).

### Real-time qPCR

All steps were performed in a designated PCR-clean area. RNA was extracted from the treated THP-1 macrophages using the peqGOLD total RNA kit (PeqLab, Austria) as specified by the manufacturer. cDNA was synthesized with 1 μg of total RNA reverse-transcribed using oligo (dT) and MultiScribe Reverse Transcriptase. For quantitative PCR, cDNA was added to a 15 μl reaction using LightCycler 480 SYBR Green I Master kit (Roche, Germany). Relative gene expression was calculated as the ratio of the detected expression of each gene normalized to that of 18S. The used primers (QuantiTect Primer Assay; Qiagen, Germany) are Hs_ABCA1_1_SG (QT00064869), Hs_ABCG1_1_SG (QT00021035), Hs_SCARB1_1_SG (QT00033488), and Hs_RRN18S_SG (QT00199367).

### Cholesterol efflux assay

Detailed information concerning the ChE assay setup can be found in previous reports (Wang et al., 2016, 2017). Briefly, THP-1 macrophages were labeled with [^3^H]-cholesterol and treated with 10 μM erythrodiol, 10 μM pioglitazone, and solvent vehicle control (DMSO) for 24 h. After incubation, cells were treated with the same compounds with and without apo A1 for further 6 h to induce ChE from macrophages. Apo A1-mediated ChE was calculated as:

$$\begin{array}{l} \text{Apo}\;\text{ A}1\;\text{mediated}\;\text{cholesterol}\;\text{efflux}\;\%\\ \;=\;(\frac{\left(\text{extracellular}\;\text{cpm}\right)\;\text{apo}\;\text{ A}1}{\left(\text{total}\;\text{cpm}\right)\;\text{apo}\;\text{ A}1}\\ \;-\;\frac{\left(\text{extracellular}\;\text{cpm}\right)\;\text{ no}\;\text{apo}\;\text{A}1}{\left(\text{total}\;\text{cpm}\right)\;\text{no}\;\text{apo}\;\text{ A}1})\times 100 \end{array}$$

### Statistical analysis

All experiments were performed at least three times. The data are presented as the mean ± *SD*. Statistical evaluation was done by one-way analysis of variance (ANOVA) using GraphPad Prism 5 software (GraphPad Software Inc.). Differences between the vehicle control group and experimental condition with a *p* < 0.05 were considered statistically significant.

## Results

### Screening of nine olive oil components for an effect on ABCA1 protein expression

**Figure 2:**
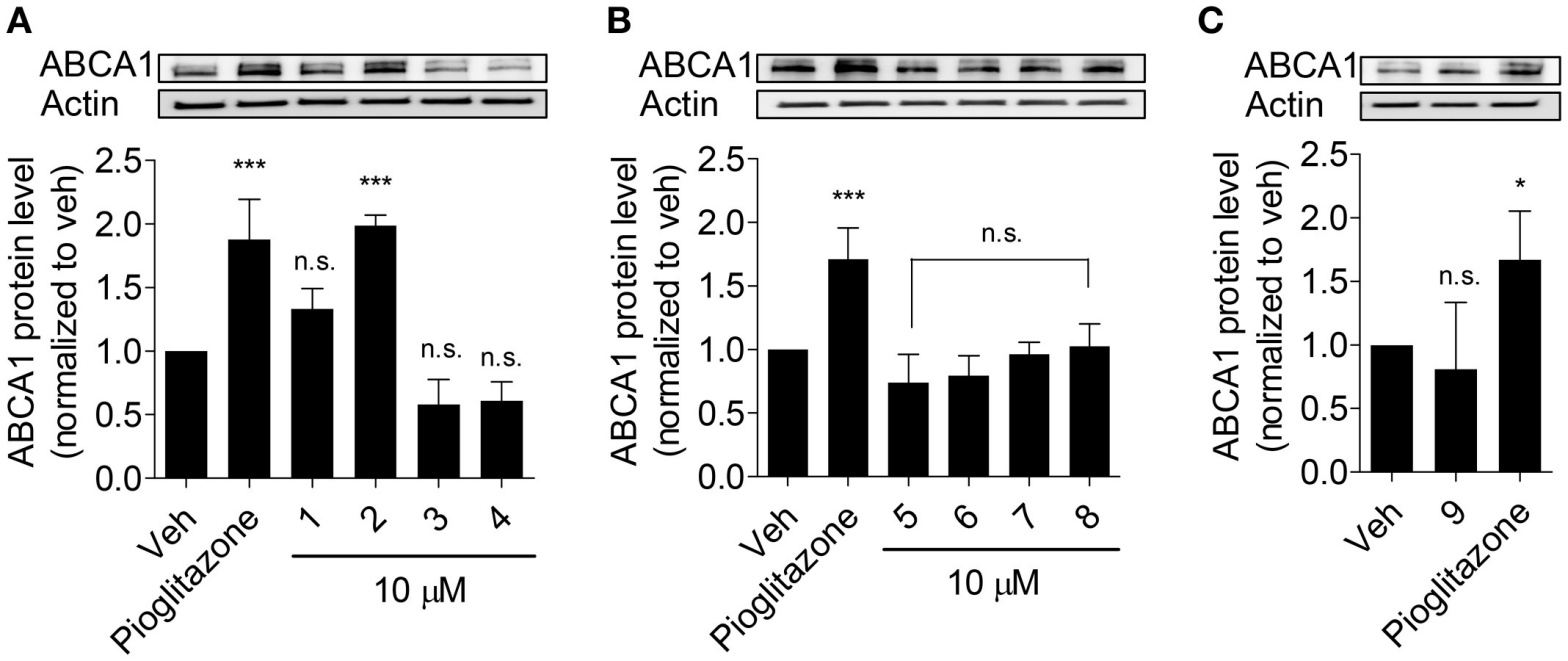
Influence of olive oil components **1**–**9** (as in Figure 1) on ABCA1 protein level **(A–C)**. Differentiated THP-1 macrophages were treated with the indicated components for 24 h. After incubation, cells were lysed and subjected to western blot analysis. Pioglitazone (10 μM) was used as positive control. Data are expressed as mean ± *SD* of three independent experiments and evaluated by one-way ANOVA analysis with the Bonferroni post-test. ^*^p < 0.05, ^***^p < 0.001 compared with solvent vehicle control (DMSO), n.s. not significant vs. DMSO.

**Figure 3:**
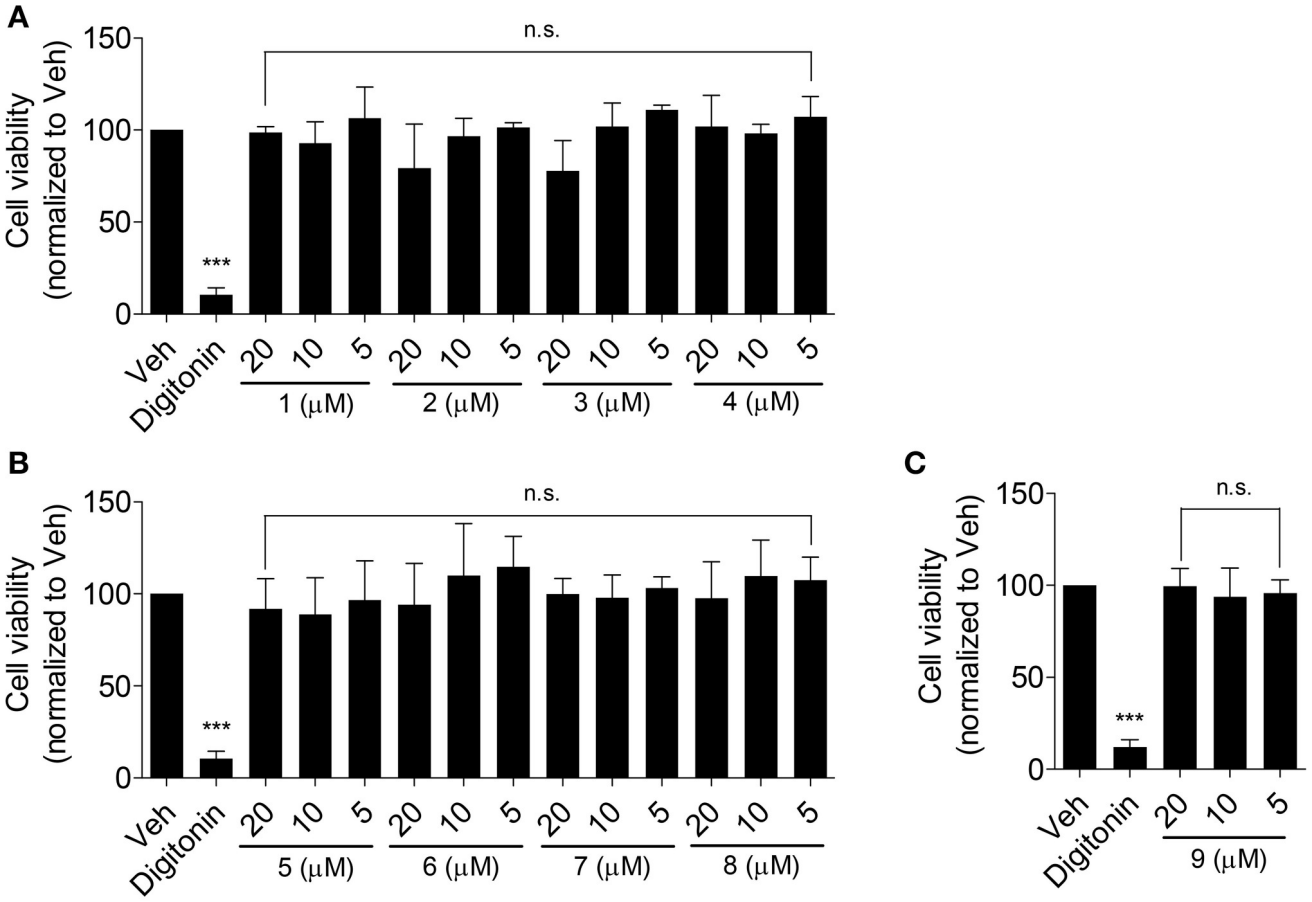
Effect of the olive oil components **1**–**9** (as in Figure 1) on the viability of THP-1-derived human macrophages **(A–C)**. THP-1 macrophages were treated with the respective compounds at the indicated concentrations for 24 h. After that, cells were washed and incubated with 10 μg/mL resazurin for further 4 h. Fluorescence derived from the converted resazurin was measured and evaluated as cell viability. A cytotoxic natural product, digitonin (at 50 μg/mL), was used as positive control. Data are expressed as mean ± *SD* of four independent experiments and evaluated by one-way ANOVA analysis with Bonferroni post-test. ^***^p < 0.001 compared with solvent vehicle control (DMSO), n.s., not significant vs. DMSO.

Olive oil components are considered an important healthy element of the Mediterranean diet (Tripoli et al., 2005). In this study, first we tested nine compounds typically present in olive oil. The compounds could be divided into two categories, pentacyclic triterpenes (uvaol, erythrodiol, maslinic acid, oleanolic acid, betulinic acid) and phenolic compounds (oleuropein, oleocanthal, 3-hydroxytyrosol, chlorogenic acid; Figure 1). All compounds were tested at 10 μM and pioglitazone, a full PPARγ agonist, was used as positive control to augment ABCA1 protein level (Ozasa et al., 2011). As shown in Figure 2, the tested compounds (10 μM) exhibited no significant effect on ABCA1 protein level, except erythrodiol, which was as effective as pioglitazone (Figure 2A). Besides, none of the compounds had a significant effect on the viability of THP-1 macrophages at 5, 10, and 20 μM, respectively, as shown by the resazurin conversion assay (Figure 3).

### Erythrodiol concentration-dependently increases ABCA1 protein level and promotes cholesterol efflux from THP-1 macrophages

**Figure 4:**
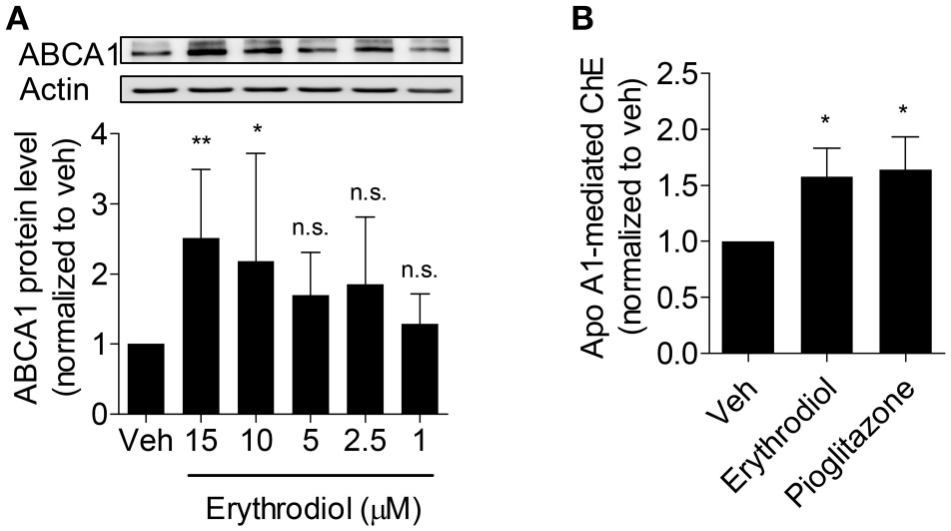
Erythrodiol concentration-dependently increases ABCA1 protein level **(A)** and enhances apo A1-mediated ChE **(B)**. **(A)** THP-1 macrophages were incubated with erythrodiol at different concentrations (1, 2.5, 5, 10, and 15 μM) for 24 h. After incubation, cells were lysed and extracted protein was applied to western blot analysis. **(B)** THP-1 macrophages were treated with solvent vehicle control (DMSO), 10 μM erythrodiol and 10 μM pioglitazone and loaded with [^3^H]-cholesterol, simultaneously. After 24 h incubation, cells were washed with PBS and incubated with the same compounds for another 6 h. At the same time, cells were divided into two groups and treated with and without apo A1. Scintillation counting was performed for radioactivity measurement. Pioglitazone was used as positive control to induce ChE. Data are expressed as mean ± *SD* of four independent experiments and evaluated by one-way ANOVA analysis with Bonferroni post-test. ^*^p < 0.05, ^**^p < 0.01 compared with DMSO, n.s., not significant vs. DMSO.

Since erythrodiol increased ABCA1 protein level at 10 μM, we further studied whether erythrodiol enhances ABCA1 protein level in a concentration-dependent manner. As expected, erythrodiol enhanced ABCA1 protein level from 1 to 15 μM and reached significance at 10 and 15 μM (Figure 4A). ABCA1 is the most important transporter protein for ChE from macrophages and effluxes cholesterol that binds to apoA1 (Duffy and Rader, 2006; Westerterp et al., 2014; Du et al., 2015). Enhanced ABCA1 protein level is expected to result in increased ChE (Wang et al., 2015). Therefore, we measured erythrodiol-induced and apoA1-mediated ChE from THP-1 macrophages. Indeed, erythrodiol at 10 μM significantly promoted ChE from THP-1 macrophages, comparable to the positive control pioglitazone, which is a well-known macrophage ChE enhancer (Figure 4B; Ozasa et al., 2011).

### Erythrodiol does neither enhance ABCG1 and SR-B1 protein level nor increase the mRNA level of ABCA1, ABCG1, and SR-B1

**Figure 5:**
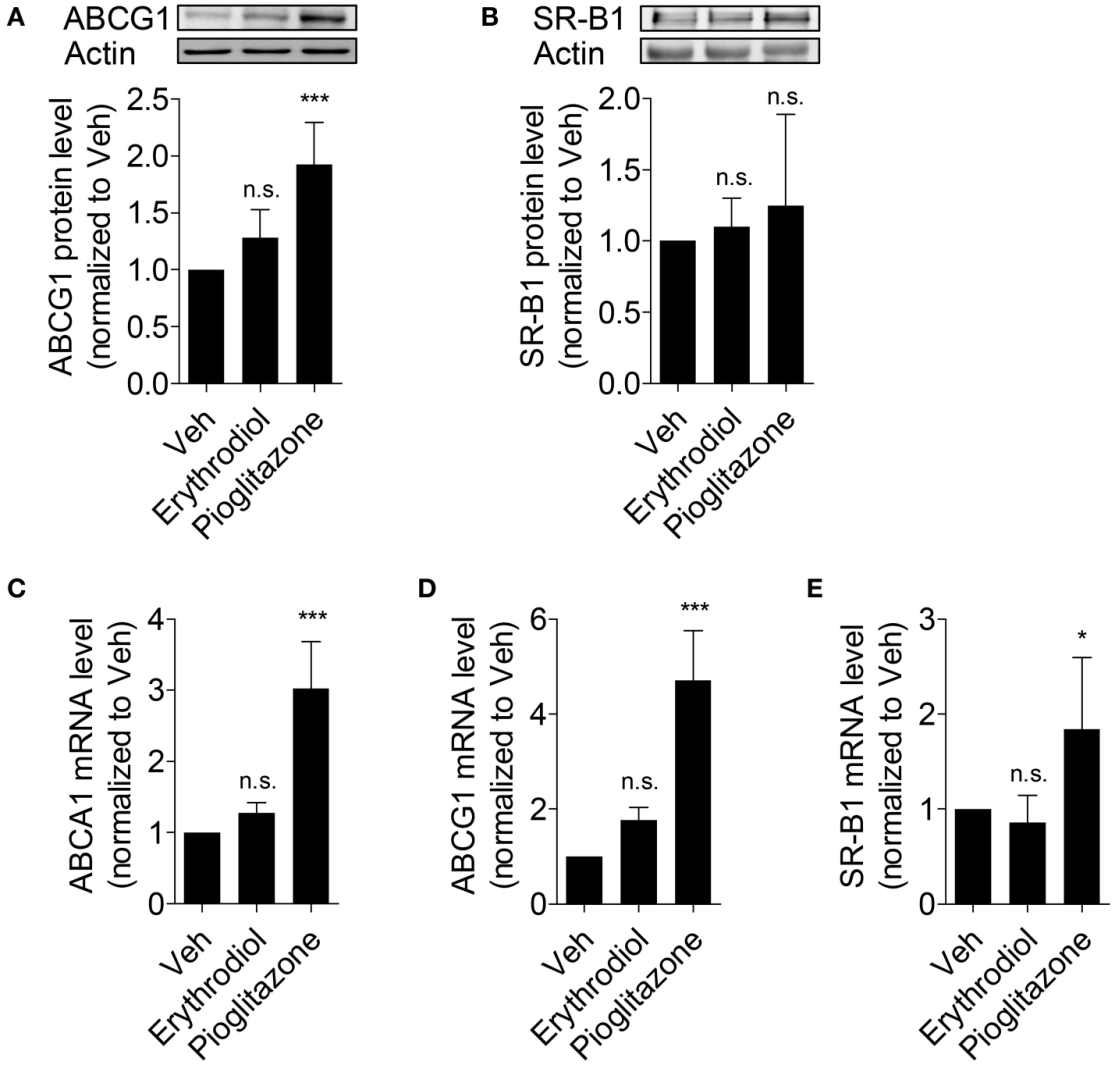
Erythrodiol has no effect on protein expression level of ABCG1 **(A)** and SR-B1 **(B)**, and fails to increase the mRNA level of ABCA1 **(C)**, ABCG1 **(D)** and SR-B1 **(E)**. THP-1 macrophages were incubated with solvent vehicle control (DMSO), erythrodiol (10 μM), and pioglitazone (10 μM) for 24 h. After incubation, cells were lysed and extracted protein was applied to western blot analysis **(A,B)**. Total RNA from the treated cells was extracted, followed by cDNA synthesis **(C–E)**. qPCR was performed and quantified based on four independent experiments. The expression level of each gene was normalized to 18S. Data are expressed as mean ± *SD* of at least three independent experiments and evaluated by one-way ANOVA analysis with Bonferroni post-test. ^*^p < 0.05, ^***^p < 0.001 compared with DMSO, n.s., not significant vs. DMSO.

Besides ABCA1, there are further two important transporters (ABCG1 and SR-B1) reported to contribute to macrophage ChE (Phillips, 2014; Westerterp et al., 2014). Since erythrodiol promotes ChE from THP-1 macrophages and increases ABCA1 protein level, the expression level of ABCG1 and SR-B1 was tested to explore the specificity of action of erythrodiol. As presented in Figure 5, the protein level of ABCG1 (Figure 5A) and SR-B1 (Figure 5B) remained unaffected. Also mRNA level of these two genes remained unchanged (Figures 5D,E). Though ABCA1 protein level increased in response to erythrodiol, its mRNA level remained unaltered (Figure 5C). The balance between their *de novo* synthesis and degradation determines the abundance of cellular proteins (Baskin and Taegtmeyer, 2011). We, therefore, tested whether the enhanced ABCA1 protein level could be due to an altered (diminished) protein degradation rate.

### Erythrodiol inhibits ABCA1 protein degradation

**Figure 6:**
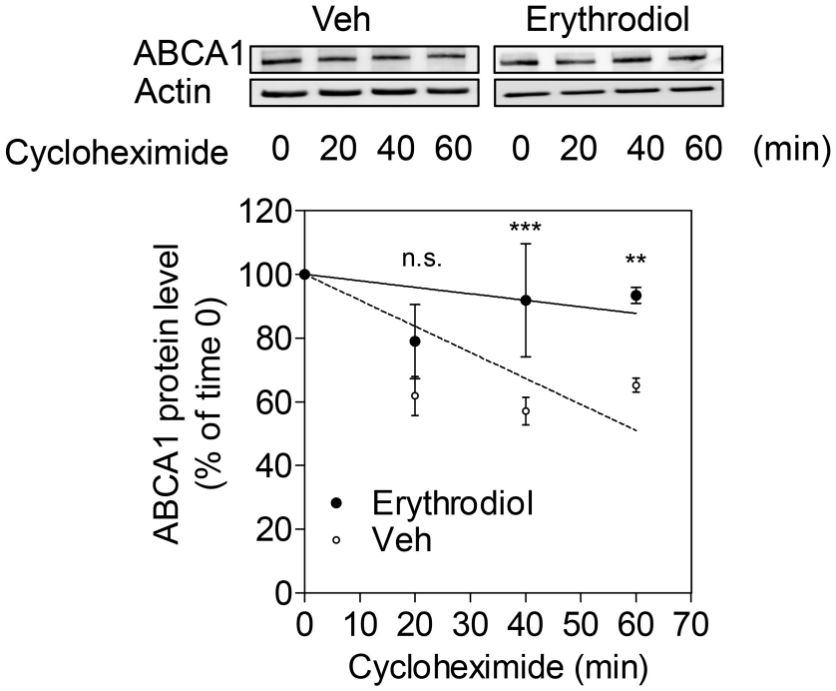
Effect of erythrodiol on the ABCA1 protein degradation rate. Differentiated THP-1 macrophages were treated with 10 μM erythrodiol or solvent vehicle control (DMSO). After 24 h incubation, cells were treated with 140 μM cycloheximide and lysed at the indicated time points (0, 20, 40, and 60 min). Total protein was extracted and subjected to Western blot analysis. Data are expressed as mean ± *SD* of three independent experiments and evaluated by two-way ANOVA analysis with Bonferroni post-test. ^**^p < 0.01, ^***^p < 0.001 compared with the solvent vehicle control (DMSO), n.s., not significant vs. DMSO.

ABCA1 protein degradation rate was tested in the presence of cycloheximide, a protein *de novo* synthesis inhibitor, by monitoring the remaining ABCA1 protein at different time points after the application of cycloheximide (0, 20, 40, 60 min). ABCA1 protein degradation was significantly inhibited by erythrodiol (10 μM; Figure 6). A first significant effect was detected 40 min after the application of cycloheximide. At 60 min, the ABCA1 protein was degraded by 50% under control condition, while around 90% of the protein was still present under the erythrodiol treatment condition. These results overall imply that erythrodiol increases ABCA1 protein level by inhibiting its degradation rather than enhancing ABCA1 gene transcription.

## Discussion

In this study, we show that the olive oil component erythrodiol concentration-dependently increases ABCA1 protein level. Our study reveals that erythrodiol promotes ChE most likely by inhibiting the degradation of ABCA1 protein, without affecting protein level of other key cholesterol transporters, i.e., ABCG1 and SR-B1. Our results may provide hints for the molecular mechanisms underlying previous observations that consumption of olive oil is associated with beneficial effects in the context of cardiovascular disease (Lou-Bonafonte et al., 2012; Helal et al., 2013; Covas et al., 2015).

Despite recent advances in studying olive oil and its components, still little is known about the compounds that mediate its cardiovascular protective effects. Based on recent randomized controlled human studies, virgin olive oil has exhibited additional benefits for human health compared to other vegetable oils in the context of cardiovascular risk factors. As reported virgin olive oil increased HDL-cholesterol level, reduced the oxidative damage to lipids, decreased inflammation, and improved endothelial function, among other beneficial effects (Covas et al., 2015). Specifically, consumption of virgin olive oil promotes HDL-mediated ChE and increases ABCA1 and ABCG1 expression in human macrophages (Helal et al., 2013). Another randomized controlled trial indicated that enhanced ChE and the increased expression of related genes (ABCA1, SR-B1, and PPARα/γ/δ) might be also attributed to olive oil polyphenols (Farras et al., 2013). However, none of the previously published reports have studied the ChE-enhancing potential of single components of olive oil extracts. Taken together, our study is the first to investigate the potential of the above mentioned compounds present in olive oil on ChE and its related ABCA1 gene expression. Surprisingly, the polyphenols oleuropein, oleocanthal, 3-hydroxytyrosol, and chlorogenic acid, did not show any effect as a single compound on ABCA1 protein expression (Figures 2B,C), which is an important determinant for ChE (Wang et al., 2015). Despite lack of effect of single polyphenol on ABCA1 expression, they nevertheless may positively affect atherosclerosis by different mechanisms, i.e., through direct inhibition of oxidation, especially of LDL, which is atherogenic after oxidative modifications (Owen et al., 2000a,b,c; Tripoli et al., 2005).

Former reports indicated anti-tumor effect of erythrodiol, particularly in the context of colon adenocarcinoma and skin tumors (Nishino et al., 1988; Juan et al., 2008). The compound was also shown to inhibit 2-*O*-tetradecanoylphorbol-13-acetate (TPA) induced inflammation in a model of chronic skin inflammation (Manez et al., 1997). Besides, erythrodiol showed additive vasorelaxant activities in aortic rings with functional endothelium pre-contracted by 10^−6^ M-phenylephrine together with oleanolic acid. The observed relaxation was mainly mediated by endothelial nitric oxide production (Rodriguez-Rodriguez et al., 2004). Taken together, our results show an additional mechanism, by which erythrodiol may protect the cardiovascular systems.

Since erythrodiol is effective in promoting ChE (Figure 4B), it may act in the context of RCT eliminating cholesterol from peripheral tissues. ChE is tightly regulated by transcriptional pathways, mainly through activation of nuclear receptors such as PPARγ, LXR, and RXR. Some dietary natural compounds are known to be able to activate these receptors (Kitareewan et al., 1996; Plat et al., 2005; Wang et al., 2014). Our results show that neither ABCA1 nor ABCG1 or SR-B1 mRNA level had been affected by erythrodiol. Thus, an influence of erythrodiol on nuclear receptors regulating ChE appears unlikely. Instead, we showed that erythrodiol acts on a post-translational level inhibiting the ABCA1 protein degradation rate.

## Conclusion

On the basis of our results, it can be stated that a triterpenic compound in olive oil, erythrodiol, increases ChE likely through inhibiting ABCA1 degradation in THP-1 macrophages. Therefore, erythrodiol is a good candidate to be further studied especially in *in vivo* studies as one of the olive oil components that contribute the cardiovascular benefits associated with olive oil consumption.

## Author contributions

LW has performed most of the experiments, analyzed data, and has written the first draft of the manuscript. SW performed experiments and analyzed data. LK, AL, EH, VD, and AA planed experimental work, analyzed data, and revised and improved the first draft. All authors have seen and agreed on the finally submitted version of the manuscript.

### Conflict of interest statement

The authors declare that the research was conducted in the absence of any commercial or financial relationships that could be construed as a potential conflict of interest.
